# Modulation of Mouse Dendritic Cells In Vitro by *Lactobacillus gasseri* Postbiotic Proteins

**DOI:** 10.1007/s12602-024-10292-6

**Published:** 2024-06-05

**Authors:** Diomira Luongo, Vincenzo De Sena, Francesco Maurano, Mauro Rossi

**Affiliations:** Institute of Food Sciences, National Research Council, Avellino, Italy

**Keywords:** Lactobacilli, Immune cells, Immunomodulation, Surface-layer proteins

## Abstract

Different lactobacilli are probiotics for their beneficial effects that confer to the host. Recently, some of these effects were associated with released metabolic products/constituents (postbiotics). In the present study, the potential immunomodulatory capacity of the probiotic *Lactobacillus gasseri* OLL2809 cell-free supernatant (sup) was investigated in murine bone marrow-derived dendritic cells (DCs). Bacteria induced significantly higher expression of all examined cytokines than those induced by the stimulatory lipopolysaccharide (LPS) itself. On the contrary, sup only induced the anti-inflammatory IL-10 similarly to LPS, whereas IL-12 and IL-6 secretions were stimulated at a lower level. Moreover, sup reduced the surface expression of the analyzed co-stimulatory markers CD40, CD80, and CD86. Treatments of sup with different digestive enzymes indicated the proteinaceous nature of these immunomodulatory metabolites. Western blot and immunoadsorption analyzes revealed cross-reactivity of sup with the surface-layer proteins (SLPs) isolated from OLL2809. Therefore, we directly tested the ability of OLL2809 SLPs to stimulate specifically cytokine expression in iDCs. Interestingly, we found that all tested cytokines were induced by SLPs and in a dose-dependent manner. In conclusion, our results highlighted distinct immune properties between *L. gasseri* OLL2809 and its metabolites, supporting the concept that bacterial viability is not an essential prerequisite to exert immunomodulatory effects.

## Introduction

Intestinal homeostasis is a finely regulated body function based on complex interactions among microbiota, the epithelial line and the underlying immune system. These interactions ensure a mutually beneficial relationship aimed to exert a pro-inflammatory response towards pathogens as well as to tolerate symbiotic microbiota and food antigens [1]. Probiotics provide a substantial contribution to mucosal homeostasis, demonstrated by suppression/inhibition of pathogens, promotion of epithelial cell survival and barrier reinforcement, and immune system maturation [2]. Recently, different beneficial effects of probiotics have been associated with factors released by bacteria, named postbiotics. In 2021, the International Scientific Association of Probiotics and Prebiotics defined postbiotics as a “preparation of inanimate microorganisms and/or their components that confers a health benefit on the host” [3]. Secreted postbiotics exhibit numerous beneficial effects: antiviral [4], anti-inflammatory [5], instrumental for maintenance of intestinal integrity [6, 7]. In addition to secreted metabolites, released macromolecules from the cell surface of probiotic bacteria play a role in mediating different beneficial effects toward the host [8, 9]. Several studies have focused on the surface layer proteins (SLPs), a major factor in intestinal adhesion for lactobacilli [10, 11]. SlpA of *L. acidophilus* NCFM has been shown to bind dendritic cell (DC) C-type lectin receptors [12] and exert immunomodulatory signals which mitigate inflammatory disease states and promote maintenance of a healthy intestinal barrier function [13]. More recently, Chandhni and collaborators [14] highlighted the beneficial role played by SLPs derived from *L. plantarum MTCC 5690*, *L. fermentum MTCC 5689*, and L. *acidophilus NCFM* in a colitis mouse model. *Lactobacillus gasseri* OLL2809 is a bacterial microorganism, isolated from human feces, whose probiotic abilities have been shown by several studies [15] as well as its ability of preventing food allergies [16]. In a murine endometriosis model, this strain was effective in preventing the growth of endometrial tissue [17]. Moreover, a clinical study suggested that tablets containing OLL2809 contributed to improving the quality of life in patients with endometriosis [18]. We have previously reported that γ-irradiated *L. gasseri* cells were able to modulate the function and the phenotype of murine dendritic cells (DCs) [19, 20]. DCs play critical roles in activating innate immune cells and initiating adaptive immune responses [21]. DCs regularly interact with intestinal bacteria, extruding the dendrites through the intestinal epithelium, but the molecular mechanisms underlying these interactions are not completely elucidated. The aim of the present work was to further characterize the probiotic properties of *L.gasseri* OLL2809 investigating the potential immunomodulatory ability of its metabolites in DC activation and to identify the bacterial factors responsible for this immunomodulatory activity.

## Materials and Methods

### Bacterial Strains and Culture Conditions

*Lactobacillus gasseri* OLL2809 is a probiotic strain [16]. Cultures were routinely grown in de Man Rogosa Sharpe (MRS) broth (Difco, Detroit, Michigan, USA) for 24 h at 37 °C under microaerobiosis. Bacteria concentration was evaluated spectrophotometrically (OD_600_) by converting the detected absorbance in the corresponding CFU/mL value, using a previously established conversion factor. Then, bacteria were γ-irradiated (2800 Gy) to block proliferation and plated in a 12-well plate at a concentration of 3 × 10^6^ CFU/well using X-Vivo 15 medium for 24 h. Culture medium was collected and centrifuged at 9000 g 10 min at 4 °C, and the supernatant was ultrafiltered (cut off 0.2 μm). The cell-free supernatant (sup) was immediately used or stored in aliquots at −80 °C.

### Isolation and In Vitro Challenge of Murine Bone Marrow-Derived Dendritic Cells

C57/BL6 mice were maintained under pathogen-free conditions on a standard diet at the animal facility of the Institute of Food Sciences (accreditation no. DM.161/99). To generate dendritic cells according to a well-established protocol [22], one 6-week old mouse was euthanized by inhalation of 5% isoflurane solution, in accordance with the review committee of the Health Ministry, General Division of Animal Health-Veterinary Medicine, and EU Directive 2010/63/EU. Then, both femurs and tibiae were dissected in sterile conditions and bone marrow cells were flushed in RPMI 1640 medium supplemented with 25 mM HEPES, penicillin 100 IU/mL, streptomycin 100 IU/mL, and 10% fetal calf serum (complete medium; Lonza-Euroclone, Pero-MI, Italy). Collected cells were resuspended at a concentration of 2 × 10^5^/mL in a complete medium containing 20 ng/mL granulocyte-macrophage colony-stimulating factor **(**ImmunoTools GmbH; Friesoythe, Germany) (culture medium). Ten milliliter of the cell suspension was seeded in 100-mm Petri dishes. Ten milliliter of fresh culture medium was added on day 3, followed by changes of half-volume culture medium on days 5 and 7. On day 9, non-adherent cells, corresponding to the immature DCs (iDCs) were harvested, centrifuged, and resuspended in X-Vivo 15 medium (X-Vivo) (Lonza-Euroclone). For in vitro challenge, experiments cells (2 × 10^6^/mL) were placed in 12-well plates (1.5 ml/well) and incubated with irradiated bacteria (30:1, bacteria:DC ratio) or resuspended in sup (1.5 ml/well) for 24 h. In some experiments, iDCs were pulsed with lipopolysaccharide (LPS) for 6 h (LPS pulse) at different doses to induce DC maturation (mDCs). Cell viability was microscopically evaluated by dye-exclusion test using Nigrosin (1% solution). Cultures were centrifuged at 1000 g 10 min at 4 °C to collect cells for FACS analysis. Supernatants were further centrifuged at 10,000 g 10 min at 4 °C to eliminate any debris and the cell-free supernatants were stored at −80 °C for cytokine analysis.

### Physical and Enzyme Treatments of Microbial Metabolites

In some experiments, aliquots of sup (1.5 mL) were subjected to different treatments before in vitro challenge. For heat inactivation, aliquots were incubated for 10 min at 90 °C. For proteinase K treatment, aliquots were incubated for 60 min at 37 °C with 50 µg/mL enzyme (600 mAnsonU/mL; Euroclone) followed by 10 min enzyme inactivation at 90 °C. For RNase treatment, aliquots were incubated for 60 min at 37 °C with 50 µg/mL Rnase A, DNase-free (≥ 30 units/mg protein; Hoffmann-La Roche, Basel, Switzerland). For DNase treatment, aliquots were incubated for 60 min at 37 °C with 8 µg/mL Dnase I (3283 units/mg protein; Hoffmann-La Roche).

### FACS Analysis

DCs were stained with phycoerythrin (PE)- or fluorescein isothiocyanate (FITC)-conjugated Abs (BioLegend, San Diego, CA, USA) against mouse CD11b, CD11c, CD40, CD86, and CD80 molecules. Cell staining was analyzed using a CyFlow Space flow cytometer (Partec, Munster, Germany) and FlowJo software (Tree Star Inc., Ashland, OR, USA). For each specific antibody, a matched subclass isotype control was used.

### Cytokine Profile

Culture supernatants were quantitatively analyzed for secreted IL-12, IL-6, and IL-10 by sandwich-type ELISA. 96-well plates (Nunc Maxisorb; eBioscience Inc., San Diego, CA) were used. Capture antibody solution (100 µl/well; BioLegend) was added and incubated overnight at 4 °C. The solution was removed and 200 µl/well of blocking buffer (1% BSA in PBS) was added for 2 h at room temperature. The buffer was then removed, and 100 µl/well solution containing dilutions of standard in 0.05% Tween-20 in blocking buffer or undiluted samples was added and incubated overnight at 4 °C. After three washing steps with 0.05% Tween-20 in PBS, 100 µl of biotinylated antibody solution was added for 2 h at room temperature. After another three washing steps, 100 µl streptavidin–horseradish peroxidase conjugate (1:1500 dilution; BioLegend) was added to the wells and incubated for 1 h at room temperature. Then, after final washing, 200 µl/well revealing solution, consisting of 0.66 mg/mL o-phenylenediamine/HCl and 0.05% hydrogen peroxide in buffer (63 mM Na_2_HPO_4_, 29 mM citric acid; pH 6.0) was added. Absorbance at OD_450_ was detected after 30 min incubation in the dark at room temperature by a microplate reader (model 600, Bio-Rad).

### Extraction of Surface-Layer Proteins from *L. gasseri *OLL2809

A slightly modified version of the protocol from Johnson et al. [23] was applied. *L. gasseri* OLL2809 cells were harvested in log phase from a 2 L culture by centrifugation at 2300 g for 10 min at 4 °C. The recovered cell pellet was extensively washed with cold PBS and resuspended in 25 mL 5 M LiCl. The suspension was stirred at 150 rpm overnight at 4 °C and then centrifuged at 8000 g for 10 min at 4 °C. The supernatant was collected and dialyzed against cold distilled water 24 h (membrane cut off, 6000–8000 kDa) to form a precipitate containing the purified surface-layer proteins (SLPs). Proteins were collected by centrifugation at 18,000 g for 60 min and stored at −80 °C before use.

### Immunoadsorption of Metabolites

Purified SLPs were used to produce an in-house hyperimmune mouse anti-serum by parenteral immunization. Serum IgG from immunized or untreated mice was adsorbed on Protein-A Sepharose by using the Affi-Prep^®^ Protein A MAPS^®^ II Kit (Bio-Rad) according to the manufacturer’s instructions. Sup aliquots (1.5 mL) were incubated with 200 µl anti-SLPs or control resin for 12 h by continuous mixing at 4 °C. Samples were centrifuged at 10,000 g. Immunoadsorbed proteins were subjected to SDS-PAGE and Western blot analysis.

### SDS-PAGE and Western Blot Analysis

Protein aliquots were fractionated by 12% SDS-PAGE, detected by Coomassie R-250 staining or blotted onto ImmobilonTM PVDF membranes (Millipore, Billerica, MA, USA) and then probed with the mouse anti-SLPs serum (1:10000 dilution). After washing, the membranes were incubated with peroxidase-conjugated antibodies against mouse IgG (Dako SpA, Milano, Italy, 1:4000 dilution). Finally, immunodetection was performed using Hyperfilm™ ECL reagent (Amersham-GE Healthcare Eu-rope GmbH, Glattbrugg, Switzerland).

### Statistical Analysis

Statistical significance was determined by *t*-test or ANOVA using the GraphPad PRISM 4.0 software (GraphPad Software, Inc., La Jolla, CA). A *p*-value of 0.05 or less was considered to be significant.

## Results

### Dendritic Cell Maturation Conditions in Serum-Free Medium

**Fig. 1:**
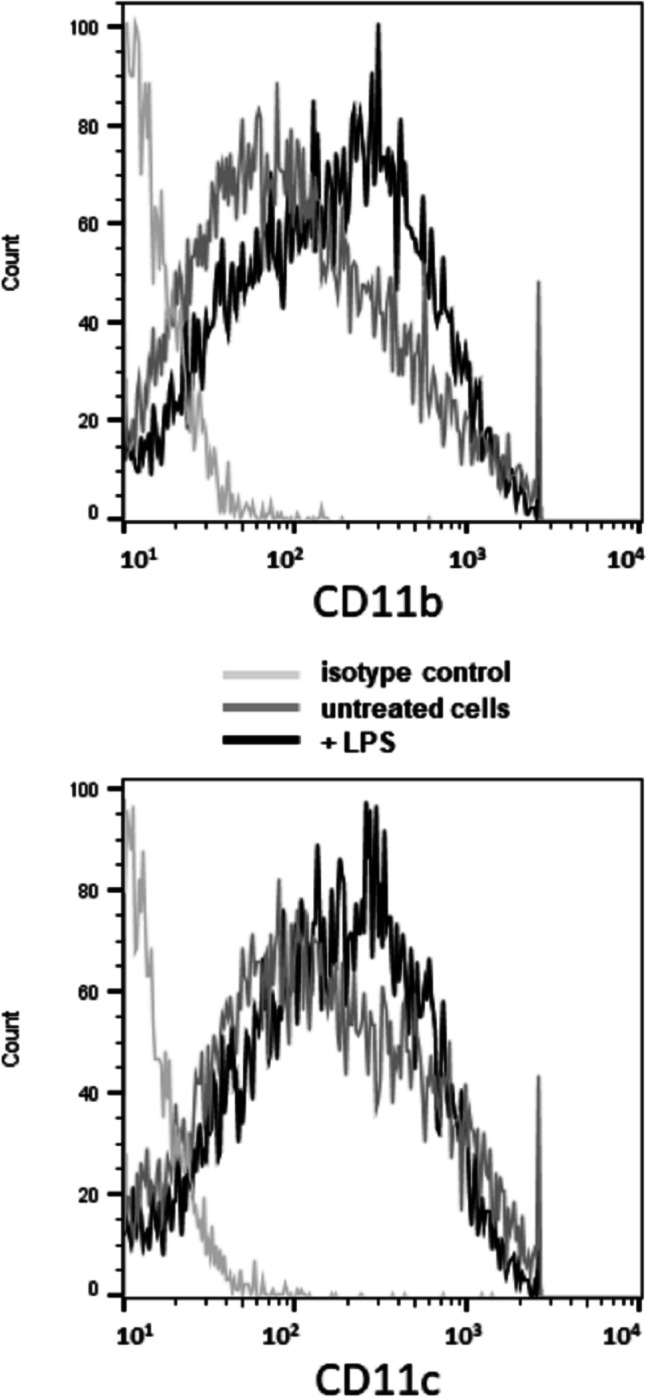
Dendritic cell phenotype. Bone marrow cells were grown in RPMI complete medium containing 20 ng/mL granulocyte-macrophage colony-stimulating factor. On day 9, non-adherent cells were harvested, resuspended in X-Vivo 15 medium (X-Vivo), and treated or not with LPS (1 µg/mL) for 6 h. After an additional 24 h incubation in X-Vivo, cells were collected and analyzed for expression of CD11b and CD11c surface markers by FACS analysis. Values are representative of three independent experiments

**Fig. 2:**
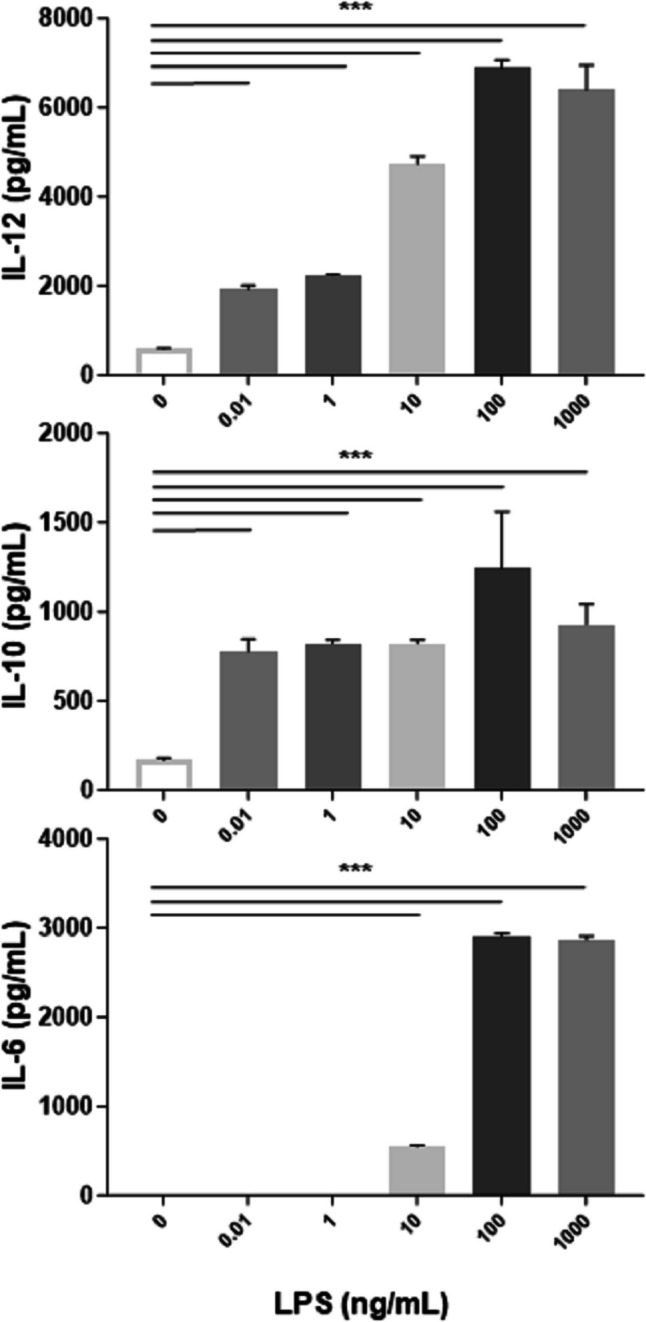
Effects of increasing lipopolysaccharide (LPS) concentrations on cytokine production by DCs. Cells were treated with increasing LPS concentrations (0.01 ng/mL to 1 ug/mL range) for 6 h (LPS pulse) and cultured for additional 24 h in X-Vivo. Culture supernatants were collected and analyzed for IL-12, IL-10, and IL-6 production by sandwich-type ELISA. Values are expressed in pg/mL. Columns represent the mean ± SD and are representative of three independent experiments. The statistical significance (***p < 0.001) is reported (ANOVA)

In order to identify immunomodulatory metabolites of *L.gasseri* OLL2809, we adopted the serum-free culture medium X-Vivo 15 (X-Vivo). iDCs normally grew and matured following LPS treatment in X-Vivo [24]. FACS analysis revealed that they are CD11b^+^CD11c^+^ DCs (Fig. 1). Importantly, lactobacilli strains survived in X-Vivo and modulated the activity of both iDCs and mDCs [24]. Thanks to the absence in X-Vivo of serum LPS-binding proteins that inhibit LPS stimulation [25] we fine-tuned further the assay conditions by identifying the minimal dose of LPS that is able to induce DC maturation. This is instrumental to unmask activities of metabolites that are present in low amount. Therefore, we pulsed iDCs with LPS encompassing the 0.01–1000 ng/mL concentration range and assessed the secretion of interleukin IL-12, IL-6, and IL-10, which represent hallmarks of DC maturation. As shown in Fig. 2, we could induce iDCs in X-Vivo by very low LPS quantities and registered a dose-response effect in the selected range for all the examined cytokines. For subsequent experiments, we used 100 ng/mL as LPS reference dose to mature iDCs.

### Differential Effects of *L. gasseri* Bacterium and Its Metabolites on Dendritic Cells

**Fig. 3:**
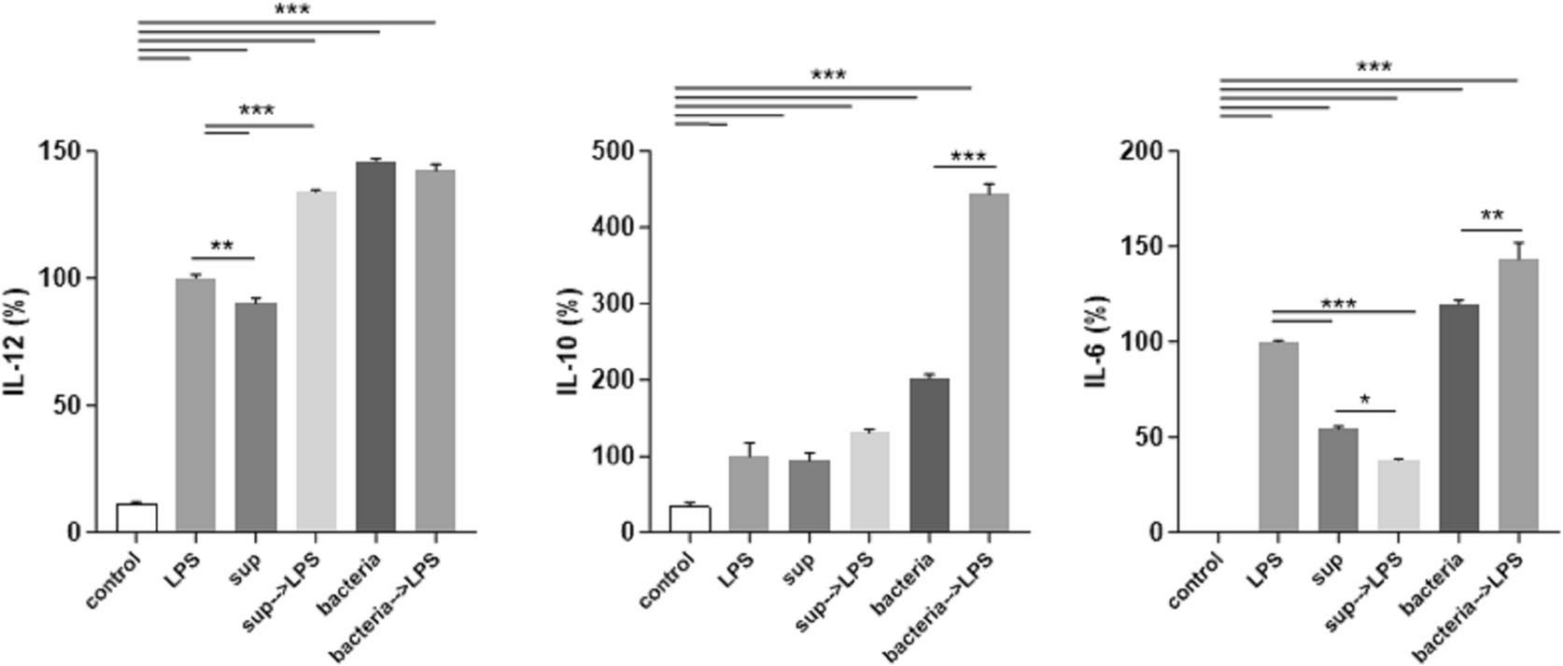
Cytokine production by iDCs and mDCs in response to irradiated *L. gasseri* OLL2809 (bacteria) or its cell-free supernatant (sup). Cytokine levels were measured after DCs were incubated for 24 h with L. *gasseri* at a 30:1 (bacteria:cell) ratio and with its sup in X-Vivo. Following incubation, some cells were treated with 0.1 µg/mL LPS for 6 h (LPS pulse) to induce the maturation of iDCs, and then all cells were cultured for additional 24 h in X-Vivo. Culture supernatants were analyzed for IL-12, IL-6, and IL-10 production by sandwich-type ELISA, and values are expressed as percentages of the maximal fluorescence intensity. Columns represent the mean ± SD and are representative of three independent experiments. The statistical significance (*p < 0.05; **p < 0.01; ***p < 0.001) is reported (ANOVA)

**Fig. 4:**
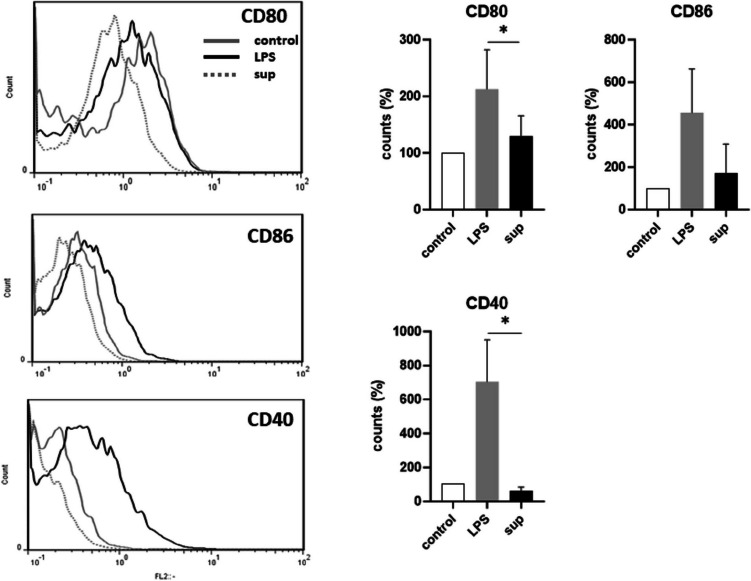
FACS analysis of co-stimulatory molecules expressed on the surface of DCs. iDCs were subjected to a 6-h LPS pulse to induce maturation or to 24 h incubation with sup and then cultured for additional 24 h in X-Vivo. iDCs were used as controls. DCs were stained for CD80, CD86, and CD40 and analyzed by FACS. Data were collected from ungated cells and are representative of three independent experiments

Next, we evaluated the effects of DC challenge with culture supernatant (sup) on the inducible cytokine profile. In control experiments, iDCs were incubated with irradiated bacteria. Bacteria induced significantly higher expression of all examined cytokines than those induced by LPS itself (Fig. 3). Bacteria pre-incubation was inductive also on mDCs (bacteria→LPS) but only for IL-6 and IL-10. When iDCs were incubated for 24 h with sup, the levels of IL-12 and IL-6 increased but at a lower degree in comparison with LPS (Fig. 3). On the contrary, sup induced the anti-inflammatory IL-10 similarly to LPS. In mDCs pre-incubation with sup (Sup→LPS) only upregulated the expression of IL-12 at levels comparable to those reported with bacteria. DC maturation is also characterized by increased expression of co-stimulatory molecules like CD40, CD80, and CD86. In our in vitro system, DC maturation was characterized by an increased expression of both markers (Fig. 4). Interestingly, bacterial metabolites markedly reduced their expression. Co-incubation with bacteria or its supernatant did not decrease DCs viability, as evaluated by measuring LDH release in the culture medium (data not shown).

### Analysis of Immunostimulatory Factors Released in the Bacterial Supernatant

**Fig. 5:**
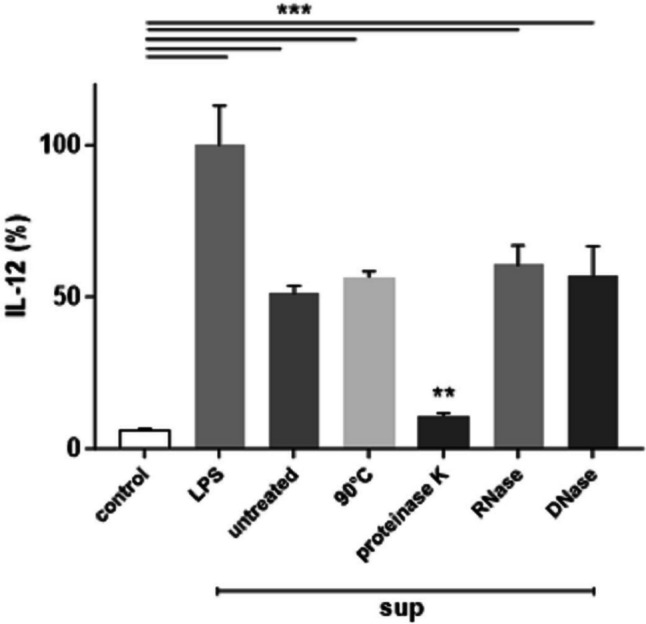
Effects of the heat and enzyme treatments of sup. iDCs were incubated for 24 h with sup pre-heated at 90 °C or digested with different enzymes (proteinase K, DNase, RNase) in X-Vivo. Culture supernatants were analyzed after further 24 h for IL-12 production by sandwich-type ELISA, and values are expressed as % of the LPS-induced cytokine. Columns represent the mean ± SD and are representative of two independent experiments. **p < 0.01 (ANOVA)

In order to characterize chemically the metabolites responsible for the reported immunostimulatory activity, sup was heat-treated (90 °C 3 min) or subjected to enzymatic treatments with proteinase K, DNase, or RNase before challenge. As a reference analyte, we selected IL-12 that has provided a higher response in our culture condition (Fig. 2). As shown in Fig. 5, only proteinase k treatment significantly reduced sup induction of IL-12. Therefore, the metabolites that showed immunostimulatory activity have a proteinaceous nature and they were still active after denaturation. To exclude any non-specific effect on cells by treatment with proteinase K, we pulsed challenged iDCs with LPS. Cytokine expression following incubation with LPS was completely restored, proving that IL-12 decrease only arose by enzyme activity (data not shown).

### Release of Surface Layers Proteins by *L. gasseri i*n X-Vivo 15 Medium

**Fig. 6:**
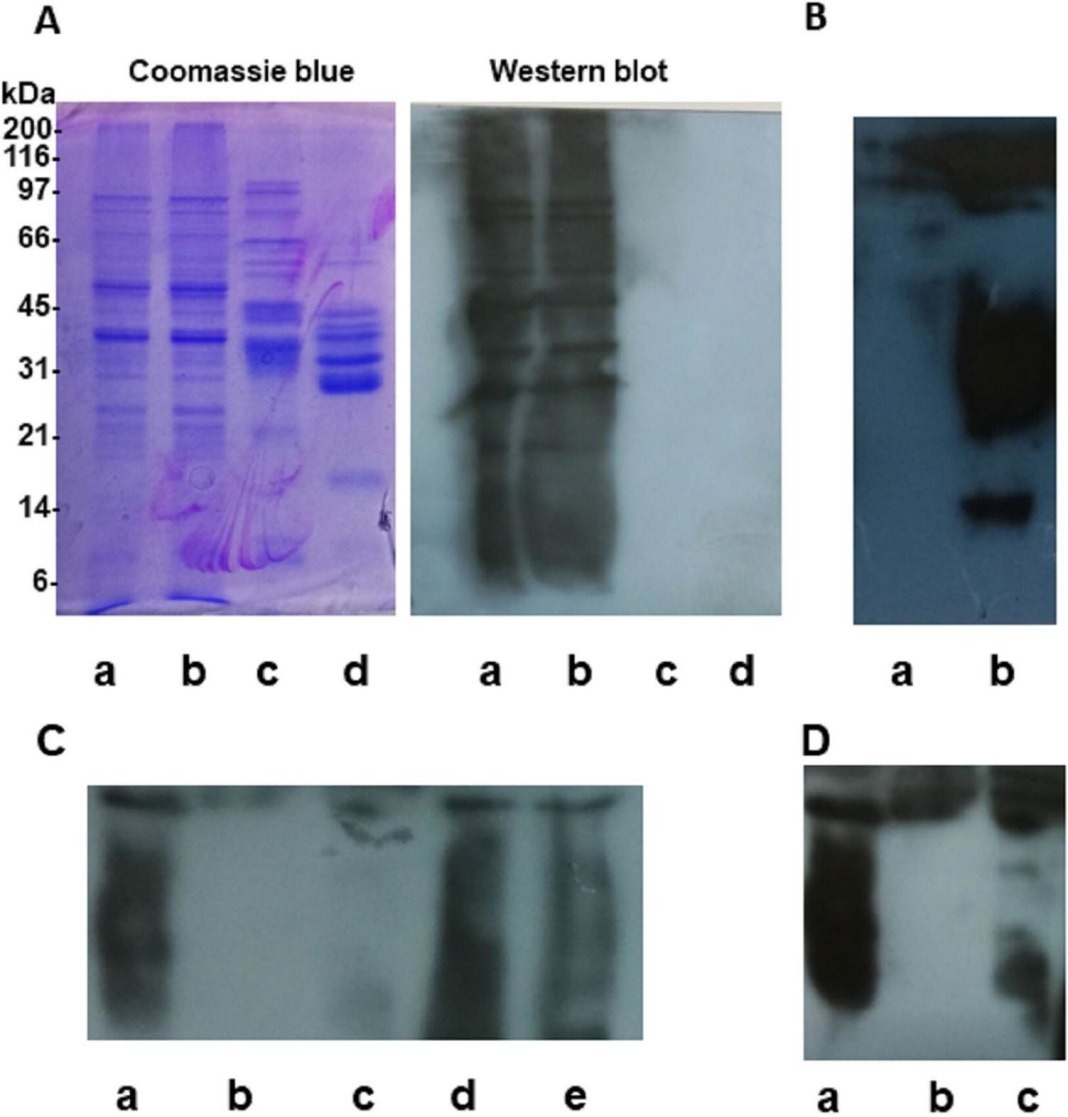
**A** Electrophoretic analysis of surface-layer proteins (SLPs) from *L.gasseri* OLL2809 by Coomassie blue staining (left panel) and Western blot (right panel) probed with anti-SLPs polyclonal antibodies. Lanes: SLPs 25 µg (a); SLPs 50 µg (b); durum wheat gliadin, 50 µg (negative control) (c); common wheat gliadin 50 µg (negative control) (d). **B** Western blot analysis of X-Vivo supernatant (sup) from a *L.gasseri* 24 h culture. Lanes: X-Vivo medium (a); sup (b). **C**, **D** Western blot analysis of SLPs in sup. sup was immunoadsorbed with anti-SLPs-IgG-Sepharose or normal serum-IgG-Sepharose. Bound proteins were eluted and subjected to SDS-PAGE and Western blot as in (**B**). The same membrane was exposed for 15 s (**C**) and 30 s (**D**) to highlight the IgG binding specificity. Lanes: untreated sup (a); eluted sample from normal serum-IgG-Sepharose (b); eluted sample from anti-SLPs-IgG-Sepharose (c);  sup following treatment with normal serum-IgG-Sepharose (d); sup following treatment with anti-SLPs-IgG-Sepharose (e)

**Fig. 7:**
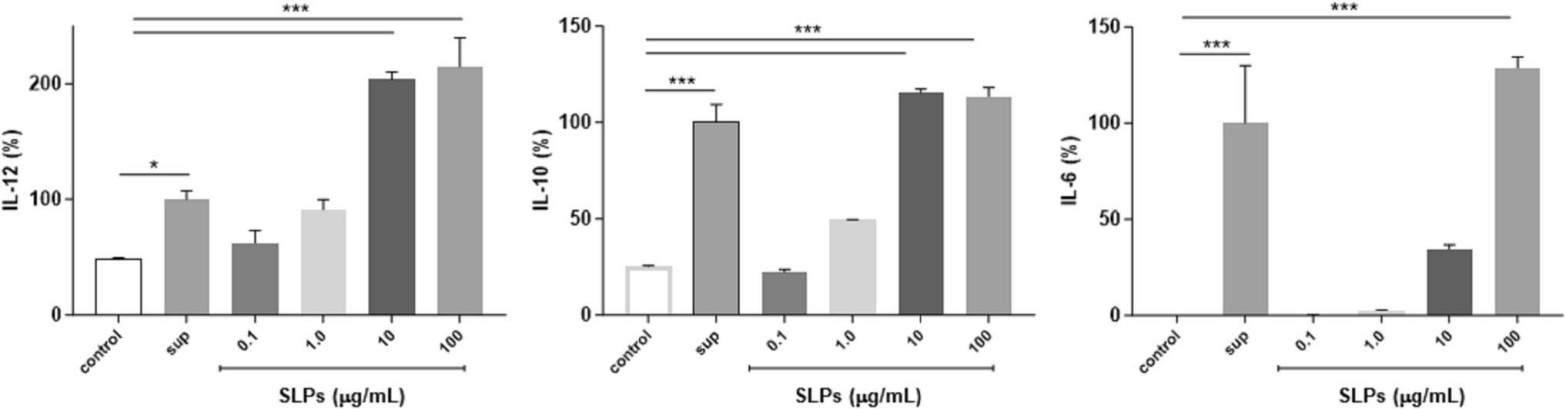
Cytokine production by iDCs in response to purified SLPs from *L.gasseri* OLL2809. iDCs were incubated with sup (positive control) or with increasing concentrations of SLPs (from 0.1 µg to 100 µg/mL) for 24 h and cultured for additional 24 h in X-Vivo. Culture supernatants were collected and analyzed for IL-12, IL-10, and IL-6 production by sandwich-type ELISA. Values are expressed as % of the sup-induced cytokine. Columns represent the mean ± SD and are representative of two independent experiments. The statistical significance (*p < 0.05; ***p < 0.001) is reported (ANOVA)

Among protein metabolites, it has been shown that surface layer proteins (SLPs) can play a role in cell function and immunomodulatory interaction [23]. SLPs represent the outermost extracellular protein component of bacteria [26]. Therefore, their presence as released proteins in sup might be envisaged. In order to detect SLPs in sup, we performed a Western blot analysis of sup by probing with a hyperimmune serum raised toward SLPs purified from OLL2809 (see Material and method section). SDS-PAGE analysis of purified SLPs revealed proteins ranging from 25 to 70 kDa with the predominant bands around 45 kDa. This latter finding was in line with previous data on surface-layer-associated proteins in lactobacilli [27]. A specific cross-reactivity of SLP proteins was reported when they were probed with a mouse polyclonal immune serum produced in-house (Fig. 6A). Notably, Western blot analysis of sup revealed the presence of different proteins cross-reacting with the anti-SLP serum (Fig. 6B). To further confirm the presence of SLPs, sup was adsorbed with anti-SLPs-IgG or normal serum as control. Immunoadsorbed proteins were recovered and subjected to SDS-PAGE and Western blot analysis. As shown in Fig. 6C, D, SLPs were specifically absorbed from sup. To evaluate whether purified SLPs showed the same inductive effect of sup on cytokines, iDCs were incubated with increasing concentrations of purified SLPs (0.1–100 µg/mL range) for 24 h and cultured in X-Vivo 15 medium for additional 24 h. We showed that all cytokines were induced by SLPs and in a dose-dependent manner (Fig. 6). Noteworthy, whereas 1 µg/mL was sufficient to induce IL-12 secretion, 10 and 100 µg/mL SLPs were needed to stimulate IL-10 and IL-6 levels comparable with those induced by sup. (Fig. 7).

## Discussion

Our in vitro study highlighted the ability of surface layers proteins isolated from *L. gasseri* OLL2809 to stimulate specifically the cytokine expression in mouse dendritic cells. Among the beneficial effects linked to the consumption of probiotics, one of the most relevant is the ability to modulate the host immune system. This aspect well explains the growing number of studies aimed at understanding the molecular mechanisms involved in the complex interaction between bacteria and the mucosal immune system. Furthermore, in recent years there has been also a shift of interest from probiotics to their cognate metabolites, named postbiotics. Herein, we addressed this issue by focusing on *L. gasseri* strain OLL2809, considering its previously reported peculiar immune-modulatory activity [19, 20]. In particular, we further characterized its biological properties in this study by investigating whether these properties could be specifically assigned to factors released in its spent medium. Considering that serum constituents could affect bacterial metabolites [28], a serum-free medium, X-Vivo 15 (X-Vivo), was adopted in this study. The use of this medium has several advantages. First, it makes the isolation and identification of candidate metabolites easier. Furthermore, the lack of serum means that its components can neither hinder nor favor the potential action of bacterial metabolites on the function and phenotype of DCs. In fact, we found that in X-Vivo, the concentration of LPS reduced by one-tenth was still able to stimulate optimally mouse bone-marrow-derived dendritic cells (DCs). DCs are fundamental in early bacterial recognition and in conditioning the subsequent responses of T lymphocytes, playing a crucial role in both innate and adaptive immunity [29]. *L. gasseri* OLL2809 and its cell-free culture supernatant (sup) were tested in DCs by focusing on the effect of the sup on the cytokine profile and on the phenotype of mature (mDCs) and immature dendritic cells (iDCs). The results showed a strong pleiotropic immune-stimulating activity of bacteria following direct challenge of iDCs, higher than those induced by LPS pulse. In addition, we confirmed this ability also when bacteria were pre-incubated before maturation. On the contrary, we found that sup was a weak inducer, especially for IL-6, while it still induced the anti-inflammatory IL-10 in iDCs at levels comparable with LPS. In parallel, sup inhibited the enhanced expression of co-stimulatory markers CD40, CD80, and CD86, a hallmark of DC maturation. Taken together, data highlighted a more specific, not pleiotropic, modulatory activity of sup on the immune response driven by DCs. These results were confirmatory of other studies reporting anti-inflammatory effects exerted by metabolites released by lactobacilli [30, 31]. We further showed that one of the most intriguing aspects of sup was its specific inhibitory effect on the LPS-induced IL-6 production. IL-6 is an important and multifunctional cytokine that can be pro-inflammatory or anti-inflammatory depending on the receptor it binds to [32]. Our findings were also in line with previous results showing suppression of various pro-inflammatory cytokines by lactobacillus postbiotics under different experimental conditions [33–35]. We also clarified the proteinaceous nature of OLL2809 immunomodulatory metabolites that were also operative in a denatured form. Therefore, the primary structure of these protein metabolites appeared to be a sufficient requisite to exert their modulatory activity. We speculated that sup metabolites were proteins non-covalently bound to the surface membrane, as are the surface-layer proteins (SLPs) or the homologous aggregation promoting factor (Apf) proteins [36]. SLPs are an important factor in the adhesion of lactobacilli to intestinal epithelial cells [10, 11, 37]. Furthermore, the immunomodulatory potential of surface proteins is also well documented [12, 13, 23, 38, 39]. We detected in sup different proteins that cross-reacted and were immunoadsorbed with an anti-SLP serum. Notably, these purified SLPs showed the ability to induce cytokine expression in iDCs similarly to sup. It must be highlighted that in the genus Lactobacillus, S-layers have been found in several but not all species [26]. In particular, on the surface of several *L. gasseri* and *L. johnsonii* strains, surface proteins have been identified that are described as S-layer-like and named Apf proteins [36, 40]. Therefore, a more detailed chemical analysis of these bacterial metabolites is needed in order to dissect further their mechanism of action as well as their potential as nutraceuticals in food applications. In conclusion, our results highlighted distinct immune properties between *L. gasseri* OLL2809 bacterium and its sup. Finally, this study confirmed that bacterial cell viability is not an essential prerequisite to exert immunomodulatory effects and paves the way for the development of innovative nutritional strategies for healthy people and the treatment/prevention of specific diseases.

## Data Availability

Data will be made available on request.
